# Circulating MicroRNAs: A Next-Generation Clinical Biomarker for Digestive System Cancers

**DOI:** 10.3390/ijms17091459

**Published:** 2016-09-01

**Authors:** Tsutomu Kawaguchi, Shuhei Komatsu, Daisuke Ichikawa, Masahiro Tsujiura, Hiroki Takeshita, Shoji Hirajima, Mahito Miyamae, Wataru Okajima, Takuma Ohashi, Taisuke Imamura, Jun Kiuchi, Hirotaka Konishi, Atsushi Shiozaki, Kazuma Okamoto, Eigo Otsuji

**Affiliations:** Division of Digestive Surgery, Department of Surgery, Kyoto Prefectural University of Medicine, 465 Kajii-cho, Kawaramachihirokoji, Kamigyo-ku, Kyoto 602-8566, Japan; t-kwgch@koto.kpu-m.ac.jp (T.K.); ichikawa@koto.kpu-m.ac.jp (D.I.); tsujiura@koto.kpu-m.ac.jp (M.T.); hiroki97@koto.kpu-m.ac.jp (H.T.); shoji-hi@koto.kpu-m.ac.jp (S.H.); miyamae@koto.kpu-m.ac.jp (M.M.); wokajima@koto.kpu-m.ac.jp (W.O.); t-ohashi@koto.kpu-m.ac.jp (T.O.); ksit15@koto.kpu-m.ac.jp (T.I.); kiuchi@koto.kpu-m.ac.jp (J.K.); h-koni7@koto.kpu-m.ac.jp (H.K.); shiozaki@koto.kpu-m.ac.jp (A.S.); kazuma@koto.kpu-m.ac.jp (K.O.); otsuji@koto.kpu-m.ac.jp (E.O.)

**Keywords:** circulating microRNA, non-invasive, biomarker, digestive tract cancer

## Abstract

MicroRNAs (miRNAs) are short noncoding RNAs that post-transcriptionally regulate gene expression and play important roles in various physiological and developmental processes such as oncogenic or tumor suppressive regulators. Specific miRNA expression signatures have been identified in a number of human cancers. Cell-free miRNAs have recently been stably detected in plasma and serum (circulating miRNAs), and their presence in blood has attracted the attention of researchers due to their potential as non-invasive biomarkers. Circulating miRNAs have emerged as tumor-associated biomarkers that reflect not only the existence of early-stage tumors, but also the dynamics and status of advanced stage tumors, tumor recurrence, and drug sensitivities. This methodology for liquid biopsy may provide non-invasive and reproductive biomarkers and individualized therapeutic strategies for cancer patients. We herein review the current phase of biological and clinical research on the circulating miRNAs of solid cancers, particularly digestive tract cancers, and discuss future perspectives. The present review may be beneficial for future research on miRNAs used to detect various cancers.

## 1. Introduction

Several studies have identified tumor-specific or -associated alterations in the circulating nucleic acids of patients with various cancers. Regarding microRNAs (miRNAs), Mitchell et al. first reported that circulating miRNAs had potential as new biomarkers in patients with solid cancers [1]. Circulating miRNAs, as non-invasive and reproducible biomarkers in cancer patients, have since attracted the attention of researchers. This concept, the so-called “liquid biopsy”, may provide ideal and individualized therapeutic strategies for cancer patients and facilitate the development of “precision medicine”. We herein review and describe the current phase of biological and clinical research on the circulating miRNAs of solid cancers, particularly digestive tract cancers, and discuss future perspectives.

## 2. The Biology and Detection of Circulating miRNAs

In 2008, Chim et al. identified the expression of miRNAs in human blood. They used qRT-PCR to enumerate miRNA expression levels in plasma of a placental origin that was collected from pregnant women [2]. Regarding cancers, several recent studies have demonstrated that extracellular nucleic acids, particularly miRNAs, occur not only through cell lysis such as tumor apoptosis and necrosis, but also through active secretion [1,3,4,5]. Tumor-derived endogenous miRNAs are present in blood in a very stable and detectable form that is protected from endogenous RNase activity. Arroyo et al. demonstrated that agonaute-2 (Ago-2), a key effector protein, is involved in miRNA-mediated silencing as a miRNA carrier in peripheral blood [6]. Furthermore, high-density lipoproteins (HDL) have been shown to participate in the mechanisms underlying intercellular communication involving the transport and delivery of miRNAs in human plasma [7]. Another secretory mechanism and biological function of extracellular miRNAs in peripheral blood involves exosome vesicles, which package a subset of miRNAs and release them through ceramide-dependent secretory systems, and secretory miRNAs are transferable and functional in recipient cells [3]. Circulating extracellular miRNAs are adequately protected against degradation by RNases and are stably detectable in peripheral blood in the forms of microvesicles such as exosomes and/or protein complexes including Ago-2 or HDL [3,6,7,8]. MicroRNAs in peripheral blood are not digested by RNase, nor are they degraded under other conditions such as low or high pH, extended storage conditions, boiling, and several freeze–thaw cycles. Moreover, sequence miRNAs are highly conserved in nature throughout species. In a few cases, changes in circulating miRNA levels have been associated with different diseases as well as certain biological or pathological stages. Previous studies demonstrated that miRNA levels may be measured without difficulty using various methods [8,9,10,11,12].

## 3. Circulating miRNAs as Cancer Diagnostic Tools

### 3.1. Esophageal Cancer

Zhang et al. investigated serum miRNA profiles in esophageal squamous cell carcinoma (ESCC) patients in an attempt to develop a novel diagnostic ESCC biomarker [13]. In that study, the findings of Solexa sequencing revealed that the expression levels of the serum miRNAs of 25 candidates were higher in ESCC patients than in controls. Furthermore, an RT-quantitative PCR (RT-qPCR) analysis identified the profiles of 7 serum miRNAs (miR-10a, miR-22, miR-100, miR-148b, miR-223, miR-133a, and miR-127-3p) as ESCC biomarkers with the area under the receiver operating characteristic (ROC) curve for the candidates ranging between 0.817 and 0.949, which was significantly higher than that for the conventional marker such as carcinoembryonic antigen [13]. We previously reported that miR-21 plasma levels were higher in ESCC patients, while those of miR-375 were lower, and the value of the area under the receiver-operating characteristic curve (AUC) was 0.816 for the miR-21/miR-375 ratio assay in ESCC patients [14]. High plasma levels of miR-21 in ESCC patients correlated with clinicopathological findings such as vascular invasion and recurrence [14].

Another study demonstrated that circulating miR-155 was of significant diagnostic value for esophageal cancer (ECa), as evidenced by an ROC curve area of 66%. However, Pearson’s analysis showed no correlation in relative miRNA expression levels between plasma and esophageal tissues, which suggested the different origins of circulating miRNAs distinct from tumor cell miRNAs. They concluded that circulating miR-155 in plasma may serve as a reliable, novel, non-invasive biomarker for the early diagnosis and detection of esophageal cancer [15]. We previously investigated whether miR-18a, which is located in the miR-17–92 cluster and was found to be strongly expressed in ESCC tissues, served as a non-invasive biomarker in plasma with ESCC. Our findings demonstrated that miR-18a plasma levels contribute to cancer detection and tumor monitoring in ESCC patients to a clinically satisfactory degree of sensitivity and specificity [16]. We also showed using microRNA array-based approaches that plasma miR-25 levels have potential as a clinically useful biomarker for cancer detection and the monitoring of tumor dynamics in ESCC patients [17]. Takeshita et al. identified serum miR-1246 as a novel diagnostic and prognostic marker in ESCC patients using microRNA arrays [18]. They showed that serum miR-1246 correlated with the tumor depth, nodal metastasis, and distant metastasis (TNM stage) and identified it as an independent risk factor for poor survival [18].

Sun et al. previously reported that plasma miRNA-718 levels were significantly lower in ESCC patients than in healthy controls, and were significantly higher in postoperative patients than in preoperative patients. Plasma miRNA-718 expression inversely correlated with nodal metastasis and the TNM stage. An ROC curve analysis revealed that AUCs using plasma miRNA-718 is 0.715, 0.689, and 0.620 for the detection of ESCC patients, Tis-T1, or TNM 0-I, respectively [19]. Another study on exosomal miRNAs in esophageal adenocarcinoma confirmed that a multi-biomarker panel of small noncoding RNAs (RNU6-1/miR-16-5p, miR-25-3p/miR-320a, let-7e-5p/miR-15b-5p, miR-30a-5p/miR-324-5p, and miR-17-5p/miR-194-5p) demonstrated enhanced specificity and sensitivity over single miRNA ratios to distinguish esophageal adenocarcinoma from controls and Barrett’s esophagus. This study also demonstrates the potential of exosomal miRNAs in serum as biomarkers for the detection of esophageal adenocarcinoma [20].

### 3.2. Gastric Cancer

A number of studies have been published on the use of circulating miRNAs for the diagnosis of gastric cancer (GC). We were the first to report the usefulness of circulating miRNAs as biomarkers in patients with GC. We selected four miRNAs (miR-17-5p, 21, 106a, and 106b) that were previously reported to be upregulated in GC as candidate miRNAs and analyzed their levels in plasma using RT-qPCR [21]. We also identified plasma miR-451 and miR-486 as novel screening markers using microRNA arrays on pre- and post-operative samples [22]. The AUC values of both miRNAs were high at 0.96 and 0.92 for the diagnosis of GC [22]. Moreover, we previously demonstrated that circulating miR-18a, which is located in the miR-17–92 cluster and was found to be strongly expressed in GC tissues, may be a useful biomarker for the screening of GC and monitoring tumor dynamics [23]. Genome-wide miRNA expression profiles followed by RT-qPCR assays revealed that miR-378 yielded an AUC of 0.861 with 87.5% sensitivity and 70.73% specificity. Collectively, these findings support our contention that circulating miR-378 has potential as a novel non-invasive biomarker in the detection of GC [24]. Several circulating miRNAs were identified as biomarkers for the detection and diagnosis of GC: miR-21, miR-200c, miR-421, miR-199a, miR-122, miR-192, miR-222, miR-16, miR-25, miR-92a, miR-451, miR-486-5p, miR-940, miR-223, miR-19b, miR-194, miR-141, and miR-1233, with a reasonable degree of diagnostic ability [25,26,27,28,29,30,31,32,33,34,35,36]. The precise and early detection and diagnosis of early GC and real-time evaluation or indication of the tumor dynamics of recurrent GC are critical issues in clinical settings.

### 3.3. Colorectal Cancer

Ng et al. analyzed the panels of 95 miRNAs, and were the first to show that miR-92 was significantly elevated in the plasma of patients with colorectal cancer (CRC) and that it has potential as a non-invasive molecular marker for CRC screening with sufficient sensitivity and specificity (89% and 70%, respectively) [37]. They also demonstrated that the detection of miR-92a may distinguish CRC from other gastrointestinal cancers and inflammatory bowel diseases. On the other hand, plasma miR-141 was identified as a non-invasive biomarker for the detection of CRC with distant metastasis using microRNA microarray profiling [38]. The availability of exosomal miRNAs in peripheral blood samples as biomarkers in CRC is currently being investigated. Ogata-Kawata reported that the serum exosomal levels of seven miRNAs (let-7a, miR-1229, miR-1246, miR-150, miR-21, miR-223, and miR-23a) were significantly higher in primary CRC patients, even those with early stage, than in healthy controls. Furthermore, they were significantly downregulated after the surgical resection of tumors [39]. Another group identified miR-378 in peripheral blood samples as a screening marker or follow-up marker for CRC patients [40].

### 3.4. Hepatocellular Cancer

A large number of studies have been published on the diagnostic availability of circulating miRNAs for hepatocellular carcinoma (HCC). Yamamoto et al. were the first to demonstrate that miR-500 is an “oncofetal” miRNA in liver cancer, having found that miR-500 was abundantly expressed in human liver cancer cell lines and 45% of human HCC tissues. They also confirmed that miR-500 levels were higher in the sera of HCC patients, and that diverse changes occur in miRNAs during liver development. They concluded that miR-500 is an oncofetal miRNA that is relevant to the diagnosis of human HCC [41]. Gui et al. identified miR-885-5p as a novel biomarker using real-time qPCR-based arrays [42]. They reported that miR-885-5p was significantly elevated in the sera of patients with liver pathologies such as HCC or liver cirrhosis (LC), and their findings indicated the potential of serum miRNAs as novel complementary biomarkers for the detection and assessment of liver pathologies. They also demonstrated that miR-885-5p correlated with other liver function parameters and hepatic histopathological indicators such as platelets, serum albumin, and the Scheuer grading system in patients with liver pathologies [42].

Qi et al. also reported that serum miR-122 has potential as a novel non-invasive biomarker for the detection of HCC in healthy subjects, and also as a novel biomarker for liver injury, but not specifically for the detection of HCC in chronic hepatitis B virus (HBV)-infected patients [43]. Similarly, other groups have identified several circulating miRNAs as non-invasive diagnostic biomarkers, such as miR-21, miR-122, miR-223, miR-15b, miR-130b, miR-101, miR-483, miR-125, miR-143, miR-215, miR-200, miR-939, and miR-595 [44,45,46,47,48,49,50,51,52,53,54,55,56]. On the other hand, the discrimination of early-stage HCC from other hepatic disorder statuses such as LC and chronic hepatitis B (CHB) is an important issue in clinical settings. Several groups identified circulating miRNAs, such as miR-19a, miR-195, miR-192, miR-146a, miR-148, miR-152, miR-122 and let-7b, miR-18a, miR-100, miR-145 miR-223 miR-200a, and miR-222, as non-invasive markers for discriminating HCC from other hepatic disorder statuses [56,57,58,59,60,61].

### 3.5. Pancreatic Cancer

Kong et al. investigated the expression levels of miRNAs in the sera of pancreatic ductal adenocarcinoma (PDAC) patients, chronic pancreatitis (CP) patients, and healthy individuals. They showed that miR-21 had the ability to distinguish PDAC patients from CP and healthy subjects, while miR-196a differentiated sera from patients with diseased pancreases (PDAC/CP) from those with normal pancreases. They also demonstrated that serum miR-196a expression levels were significantly higher in unresectable PDAC (stages III and IV) patients than in resectable (stages I and II) patients, and indicated the potential of serum miR-196a expression levels in predicting the prognosis in patients with PDAC [62]. Our group reported that several circulating miRNAs in pancreatic cancer (PCa) patients have potential as non-invasive biomarkers [63,64,65,66]. We identified circulating miR18a, which is located in the miR-17–92 cluster and is an oncogenic miRNA, as a promising biomarker with high diagnostic ability (AUC was 0.9369) in patients with PCa [63]. We also found that the expression levels of miR-221, miR-375, miR-223, and miR-744 in plasma have potential as biomarkers in PCa [64,65,66]. Regarding miR-223, plasma miR-223 levels discriminate the malignant potential between benign intraductal papillary mucinous neoplasms (IPMN) and malignant IPMN, as well as the progressive extent of invasiveness between malignant IPMN and pancreatic invasive ductal carcinoma (PIDC). Plasma miR-223 may be a useful biomarker for screening PCa, and also for predicting the malignant potential of IPMN and invasiveness of PCa in clinical settings [65].

Liu et al. confirmed that seven miRNA-based biomarkers (miR-20a, miR-21, miR-24, miR-25, miR-99a, miR-185, and miR-191) had high sensitivities and specificities for distinguishing the various stages of PCa from cancer-free controls and also accurately discriminated PCa patients from CP patients [67]. Li et al. demonstrated that serum miR-1290 levels distinguished patients with low-stage PCa from controls better than CA19-9 levels; higher miR-1290 levels predicted poorer outcomes among patients undergoing pancreaticoduodenectomy, and the detection of elevated circulating miR-1290 has the potential to improve the early detection of PCa [68]. Que et al. investigated serum exosomal miRNAs and showed that miR-17-5p and miR-21 levels were higher in PCa patients than in non-PCa patients and healthy controls, and also that high miR-17-5p levels correlated with metastasis and an advanced stage of PCa [69]. Several circulating miRNAs, such as miR-27a, miR-642b, miR-885-5p, miR-22, miR-21, miR-483, miR-1246, miR-4644, miR-3976, and miR-4306, have been identified as novel diagnostic markers with acceptable availability in PCa patients [70,71,72,73].

## 4. Malignant Potential, Tumor Recurrence, and Prognostic Biomarkers

### 4.1. Esophageal Cancer (ECa)

Our group previously identified the preoperative expression levels of circulating miRNAs, such as miR-21 and miR-375, as postoperative prognostic biomarkers in patients with esophageal cancer [74]. The postoperative cause-specific survival rate of patients with a high expression level of plasma miR-21 was lower than that of the low concentration group, while the high plasma miR-375 group showed better survival. Moreover, the prognosis of patients with high miR-21 and low miR-375 plasma levels was significantly poorer than that of other patients, and the presence of high miR-21 and low miR-375 levels was identified as an independent prognostic factor [74]. As described above, Takeshita et al. reported that serum miR-1246 is also a novel prognostic marker in ESCC patients [18]. Li et al. demonstrated that high expression levels of miR-21 and miR-16 in plasma correlated with shorter progression-free survival and overall survival (OS) in ESCC patients [75]. Odenthal et al. investigated serum miRNA profiles as prognostic markers in multimodality therapy for locally advanced adenocarcinomas in the gastroesophageal junction. They identified a correlation between high miR-192 and miR-222 expression levels and a high T-category, and also showed that miR-302c and miR-222 expression levels correlated with OS [76].

Another group used whole-miRNome profiling to identify prognostic serum miRNAs in esophageal adenocarcinoma and their relationship with the *Helicobacter pylori* (HP) infection status [77]. They found that 15 cell-free miRNAs (cfmiRNA) correlated with OS in HP-negative patients with esophageal adenocarcinoma. Moreover, a combined 2-cfmiRNA (low miR-3935 and high miR-4286) risk score was constructed; it showed a greater risk for worse OS than 15 individual cfmiRNAs alone [77].

### 4.2. Gastric Cancer (GC)

Valladares-Ayerbes et al. reported that higher expression levels of miR-200c in blood correlated with poor OS in GC patients [26]. We previously demonstrated that the postoperative cause-specific survival rate was significantly poorer in GC patients with high plasma miR-21 levels than in those with low levels [78]. Moreover, the incidence of vascular invasion was also slightly higher in GC patients with high miR-21 levels, and a multivariate analysis revealed that the presence of high miR-21 plasma levels was an independent prognostic factor [78]. Fu et al. investigated circulating miR-222 in plasma as a potential diagnostic and prognostic marker in GC [30]. Their prognostic analysis revealed a correlation between higher levels of circulating miR-222 levels and shorter disease-free survival and OS. Circulating miR-222 was also identified as an independent prognostic marker in the multivariate analysis [30]. Su et al. reported the potential of miR-18a as a biomarker for the detection of GC, and indicated that its upregulation is associated with an unfavorable prognosis [31].

Imaoka et al. found that miR-203 expression in serum were significantly lower in stage IV than in stage I–III of GC patients [79]. Serum miR-203 expression was significantly lower in GC patients with worse malignant potential such as a higher T stage, vessel invasion, and nodal, peritoneal, and distant metastases. Low expression of serum miR-203 correlated with poor disease-free survival and OS. A multivariate analysis identified low serum miR-203 expression as an independent predictive marker for metastasis such as nodal, peritoneal, and distant metastases and a poor prognosis GC patients [79].

### 4.3. Colorectal Cancer (CRC)

As described above, Chang et al. demonstrated that plasma miR-141 is a novel biomarker that complements carcinoembryonic antigen (CEA) in the detection of colon cancer with distant metastasis, and that high expression levels of miR-141 in plasma were associated with a poor prognosis [38]. Shivapurkar et al. identified a panel of six informative miRNAs (miR-15a, miR-103, miR-148a, miR-320a, miR-451, and miR-596) from a miRNA array analysis and validation study [80]. Hierarchical clustering of the expression levels of the six circulating miRNAs and a prognostic analysis showed that the risk of recurrence of early-stage colon cancer may be predicted by this panel of miRNAs, which are measurable in the circulation at the time of diagnosis [80]. Hur et al. reported that serum miR-203 levels were significantly upregulated in a stage-dependent manner, and also that the strong expression of miR-203 was associated with poor survival in patients with CRC [81]. Moreover, increased miR-203 serum levels indicated a high risk for a poor prognosis, as well as metastasis to the lymph nodes, liver, and peritoneum [81].

### 4.4. Hepatocellular Carcinoma (HCC)

Sugimachi et al. employed the microarray-based miRNAs expression profiling derived from exosomes in the serum of HCC patients to identify a biomarker that distinguishes between patients with and without HCC recurrence after liver transplantation (LT) [82]. They found that miR-718 showed a significantly different expression profile in the serum exosomes of HCC cases with recurrence after LT than in those without recurrence [82]. Köberle et al. demonstrated that patients with higher miR-1 and miR-122 serum levels had longer OS than those with lower miR-1 and miR-122 serum levels; however, serum miR-1 and miR-122 levels did not differ significantly between patients with HCC and LC [83]. Moreover, a Cox regression analysis revealed that miR-1 serum levels were independently associated with OS, whereas serum miR-122 levels were not [83]. Cho et al. revealed that high miR-122 expression levels and an advanced tumor stage were independent risk factors for poor OS in patients with hepatitis B virus-related HCC treated with radiofrequency ablation (RFA) [84]. Zhuang et al. identified low miR-128-2 serum levels as a favorable survival marker, and revealed that miR-128-2 was also an independent factor of OS in HCC patients [85].

### 4.5. Pancreatic Cancer (PCa)

Kong investigated the potential of serum miRNA expression levels as non-invasive markers for the prognosis of PDAC [62]. They reported that serum miR-196a expression levels were significantly higher in unresectable PDAC (stages III and IV) patients than in resectable (stages I and II) patients, and had the potential to predict the median survival time of PDAC patients [62]. As described above, serum miR-21 levels have been identified as a diagnostic marker by Liu et al. [67]. They also demonstrated that miR-21 serum levels correlated with OS in PCa [67]. We previously reported that high miR-221 plasma levels correlated with distant metastasis and a non-resectable status in PCa patients [64]. We also found that a high miR-744 plasma level correlated with lymph node metastasis and recurrence, and was an independent poor prognostic factor in PCa patients after pancreatectomy. It also contributed to poorer progression-free survival in non-operable PCa patients receiving gemcitabine-based chemotherapy [66].

## 5. Predicting Tool for Tumor Chemosensitivity

Few studies have examined the potential of circulating miRNAs as non-invasive biomarkers to predict tumor chemosensitivity until now. Multimodality therapy has recently emerged for patients with digestive cancers in order to improve their prognoses. Regarding CRC, Kjersem et al. examined plasma miRNAs as predicting markers for first-line oxaliplatin-based treatments for metastatic CRC (mCRC) patients [86]. The expression of 742 miRNAs was examined in plasma samples from 24 mCRC patients (12 responders and 12 non-responders) pre/post 5-fluorouracil (5-FU)/oxaliplatin treatment. The top differentially expressed miRNAs between chemotherapy responders and non-responders were selected for further analyses in a validation cohort of 150 patients. Three miRNAs (miR-106a, miR-484, and miR-130b) were found to be significantly differentially expressed before the treatment, and all three miRNAs were upregulated in non-responders. These findings indicate that plasma miRNAs analyzed before this treatment serve as non-invasive markers for predicting outcomes in mCRC patients treated with 5-FU and oxaliplatin-based chemotherapy [86]. Tanaka et al. identified several circulating miRNAs as predicting tools for preoperative cisplatin-based chemotherapy for ESCC patients [87,88]. They showed that the strong expression of serum miR-200c correlated with a poor response to chemotherapy, and also that it was associated with shorter progression-free survival [88]. Moreover, a multivariate analysis identified the expression of miR-200c as the most valuable prognostic factor for patients with esophageal cancer who receive neoadjuvant chemotherapy. They concluded that miR-200c serum levels may be useful for predicting responses to chemotherapy and the prognosis of patients with esophageal cancer receiving cisplatin-based neoadjuvant chemotherapy. They also identified serum miR-27a/b as a marker to predict cisplatin-based chemotherapy responses using microRNA arrays and validation studies [87]. A previous study demonstrated that high serum levels of miR-27a/b correlated inversely with responses to chemotherapy, while high miR-27b levels correlated with shorter cause-specific survival, and patients with high miR-27a levels also had slightly poorer prognoses that those with low miR-27a levels [87].

We recently reported that oncogenic miR-21 promoted chemoresistance in ESCC and served as a biomarker for predicting chemoresistance in the plasma of patients with ESCC [89]. Pretreatment plasma concentrations of miR-21 were found to be significantly higher in ESCC patients with a low histopathological response who underwent the preoperative chemotherapy regimen with cisplatin plus 5-fluorouracil than in those with a high histopathological response. A multivariate analysis revealed that a high pretreatment plasma concentration of miR-21 was an independent risk factor for chemoresistance. Regarding PCa, several studies demonstrated, using in vitro assays only, that cancer-related miRNAs detected in peripheral blood samples may contribute to chemosensitivity [90,91]. On the other hand, we reported that high miR-744 plasma levels contributed to poorer progression-free survival in non-operable PCa patients receiving gemcitabine-based chemotherapy [66]. Moreover, we confirmed that the overexpression of miR-744 in PCa cells induced significant chemoresistance to gemcitabine in vitro [66]. The use of circulating miRNAs as a predicting tool for chemotherapy responses has not yet been examined extensively, and, thus, requires further study.

## 6. Conclusions

Circulating miRNAs in digestive cancer patients may be promising biomarkers that are stable and reproducible in peripheral blood. They may be used for clinical applications in cancer management, such as tumor screening and early diagnoses, to evaluate malignant potential indicating surgical or non-surgical therapeutic efficiency, and to monitor for recurrence and tumor dynamics. Recent advances in cancer therapy have prolonged survival and provided various treatment options for cancer patients including chemotherapy and radiation. In the near future, circulating miRNAs may be surrogate markers and useful indicators in pretreatment decision-making for these multimodality therapies due to their non-invasiveness and reproducibility.

On the other hand, we should recognize several limitations of circulating miRNAs as biomarkers in cancer patients. First, one miRNA expression should not be used as a biomarker of a specific cancer, because several miRNAs show high expression not only one type of cancer but also other types of cancer and disease (Table 1). Second, some miRNAs should have opposite expression patterns depending on the cancer types (e.g., miR-200 family, Table 1). For these limitations, various miRNAs expression patterns or miRNAs signatures depending on each cancer might provide more reliable information as biomarkers. We also clarify the most reliable methodology to evaluate miRNAs expression in blood sample. Specifically, the methodology of purifying circulating miRNAs has emerged as central preoccupation of this field. Recently, exosomal small RNAs, which stably exist in blood and play a “cell-to-cell communication” role in several diseases including cancer, have attracted researchers’ interest. However, the best method for purification is still unclear. Furthermore, most of the studies described above were relatively small and mostly retrospective in nature. Therefore, larger studies with well-established methods are needed in order to verify the clinical availability of circulating miRNAs in patients with digestive cancers in clinical settings.

## Figures and Tables

**Table 1:** Candidates of circulating microRNAs as cancer biomarker.

Cancer Type	Expression Level ^1^	Cancer Diagnostic Tools	Malignant Potential, Tumor Recurrence, And Prognostic Biomarker	Predicting Tool For Chemosensitivity	References
Esophegeal cancer	High	miR-10a, miR-22, miR-100, miR-127, miR-133a, miR-148b, miR-223, miR-21, miR-155, miR-18a, miR-1246, miR-16, miR-25, miR-320a, let-7e, miR-15b	miR-21, miR-16, miR-1246, miR-192, miR-222, miR-3935, miR-4286	miR-200c, miR-27a/b, miR-21	[13,14,15,16,17,18,19,20,74,75,76,77,87,88,89]
	Low	miR-375, miR-718, miR-30a/miR-324, miR-17/miR-194	miR-375, miR-302c	-	
Gastric cancer	High	miR-17-5p, miR-21, miR-106a/b, miR-18a, miR-378, miR-451, miR-486, miR-21, miR-200c, miR-421, miR-199a, miR-122, miR-192, miR-222, miR-16, miR-25, miR-92a, miR-940, miR-223, miR-19b, miR-194	miR-200c, miR-21, miR-222, miR-18a	-	[21,22,23,24,25,26,27,28,29,30,31,32,33,34,35,36,78,79]
	Low	miR-141, miR-1233	miR-203	-	
Colorectal cancer	High	miR-92a, miR-141, let-7a, miR-1229, miR-1246, miR-150, miR-21, miR-223, miR-23a, miR-378	miR-141, miR-320, miR-596, miR-203	miR-106a, miR-484, miR-130b	[37,38,39,40,80,81,86]
	Low	-	miR-15a, miR-103, miR-148a, miR451	-	
Hepatocellular Cancer	High	miR-500, miR-885, miR-122, miR-21, miR-223, miR-15b, miR130b, miR-101, miR-483, miR-125, miR-143, miR-215, miR-939, miR-595	miR-718, miR-128-2	-	[41,42,43,44,45,46,47,48,49,50,51,52,53,54,55,56,57,58,59,60,61,82,83,84,85]
	Low	miR-200, miR-19a, miR-195	miR-1, miR-122	-	
Pancreatic cancer	High	miR-21, miR-196a, miR-18a, miR-221, miR-223, miR-20a, miR-24, miR-25, miR-99a, miR-185, miR-191, miR-1290, miR-27a, miR-642b, miR-885-5p, miR-22, miR-21, miR-483, miR-1246, miR-4644, miR-3976, and miR-4306	miR-196a, miR-21, miR-221, miR-744	miR-744	[62,63,64,65,66,67,68,69,70,71,72,73,89,90]
	Low	miR-375	-	-	

^1^ In cancer patients for cancer diagnostic tools, in patients with worse prognosis for malignant potential, tumor recurrence, and prognostic biomarker, and in patients with chemo-resistance, respectively.
